# Impact of smoking status and chronic obstructive pulmonary disease on pulmonary complications post lung cancer surgery

**DOI:** 10.1371/journal.pone.0266052

**Published:** 2022-03-29

**Authors:** Vishnu Jeganathan, Simon Knight, Matthew Bricknell, Anna Ridgers, Raymond Wong, Danny J. Brazzale, Warren R. Ruehland, Muhammad Aziz Rahman, Tracy L. Leong, Christine F. McDonald

**Affiliations:** 1 Department of Respiratory and Sleep Medicine, Austin Health, Heidelberg, Victoria, Australia; 2 Institute for Breathing and Sleep, Heidelberg, Victoria, Australia; 3 Department of Thoracic Surgery, Austin Health, Heidelberg, Victoria, Australia; 4 Faculty of Medicine, University of Melbourne, Parkville, Victoria, Australia; 5 School of Health, Federation University Australia, Berwick, Victoria, Australia; National and Kapodistrian University of Athens, GREECE

## Abstract

**Introduction:**

Smoking and chronic obstructive pulmonary disease (COPD) are associated with an increased risk of post-operative pulmonary complications (PPCs) following lung cancer resection. It remains unclear whether smoking cessation reduces this risk.

**Methods:**

Retrospective review of a large, prospectively collected database of over 1000 consecutive resections for lung cancer in a quaternary lung cancer centre over a 23-year period.

**Results:**

One thousand and thirteen patients underwent curative-intent lobectomy or pneumonectomy between 1995 and 2018. Three hundred and sixty-two patients (36%) were ex-smokers, 314 (31%) were current smokers and 111 (11%) were never smokers. A pre-operative diagnosis of COPD was present in 57% of current smokers, 57% of ex-smokers and 20% of never smokers. Just over 25% of patients experienced a PPC. PPCs were more frequent in current smokers compared to never smokers (27% vs 17%, p = 0.036), however, no difference was seen between current and ex-smokers (p = 0.412) or between never and ex-smokers (p = 0.113). Those with a diagnosis of COPD, independent of smoking status, had a higher frequency of both PPCs (65% vs 35%, p<0.01) and overall complications (60% vs 40%, p<0.01) as well as a longer length of hospital stay (10 vs 9 days, p<0.01).

**Conclusion:**

Smoking and COPD are both associated with a higher rate of PPCs post lung cancer resection. COPD, independent of smoking status, is also associated with an increased overall post-operative complication rate and length of hospital stay. An emphasis on COPD treatment optimisation, rather than smoking cessation in isolation, may help improve post-operative outcomes.

## Introduction

Lung cancer is the leading cause of cancer mortality worldwide [1]. In Australia, lung cancer is responsible for close to a fifth of cancer-related deaths, with a five-year survival rate of 17% [2]. Smoking remains the most significant modifiable risk factor in lung cancer development. Despite advances in tobacco control, data from the Australian Bureau of Statistics reveal that 13.8% of Australian adults still smoke daily [3]. In early stage non-small cell lung cancer (NSCLC), surgery remains the primary treatment modality in those who are operative candidates. The vast majority of patients who undergo lung cancer resection have smoked in the past, and many are active smokers at the time of surgery [4–6]. Several studies show that smokers, whether current or reformed, have higher rates of post-operative pulmonary complications (PPCs) after lung cancer resection than never smokers [6–8]. A fifth of all patients who undergo major surgery and experience a PPC die within 30 days of the operation compared to 0.2–3% of those who do not [9]. Additionally, developing a PPC is associated with higher rates of intensive care unit (ICU) admission and longer durations of hospital stay [10, 11].

Chronic obstructive pulmonary disease (COPD) coexists in 50–70% of patients with lung cancer [12] and is an independent risk factor for the development of lung cancer, over and above smoking [13, 14]. Smokers with COPD are twice as likely to develop lung cancer as smokers without COPD [15]. Several studies have shown that the presence of COPD is associated with a higher frequency of PPCs [16–19].

Despite the established link between smoking, COPD and PPCs after lung cancer resection, it is unclear whether reformed smokers have higher complication rates compared with those who continue to smoke [7, 20]. Furthermore, there is uncertainty regarding the optimal timing of smoking cessation prior to surgery, with potential benefits weighed against the risk of disease progression if operative management is delayed. Attitudes of thoracic surgeons are inconsistent, with no consensus on standard practice for pre-operative smoking cessation. A survey of US thoracic surgeons demonstrated that most (60%) do not require a patient to cease smoking prior to surgery, with those who do mandate cessation divided on the recommended duration of the smoking abstinence period [21]. Most US surgeons do not routinely refer to smoking cessation programs or prescribe nicotine replacement therapy prior to surgery [21]. although nicotine dependence treatment is known to be highly effective in effecting short-term cessation amongst cancer patients [22, 23].

Although international studies have examined smoking status, COPD and PPCs in patients undergoing lung cancer resection [7, 16, 24], there have been no studies to date in an Australian population, and few studies of the size and breadth of ours. In a large cohort of patients undergoing lung cancer resection in an Australian quaternary centre, our study aimed to examine the relationship between PPCs and (i) pre-operative smoking status, and (ii) pre-operative COPD diagnosis.

## Methods

A retrospective review was undertaken of a prospectively maintained database of consecutive operations performed for lung cancer resection. Data included demographic information, pre-operative lung function and post-operative complications. Approval was granted by the institutional Health Research Ethics Committee.

### Population

All patients who underwent lobectomy or pneumonectomy for lung cancer between 1995 and 2018 were included. Patients who had undergone segmentectomy or wedge resection were excluded, as were those with benign tumours. Surgery was performed by a dedicated thoracic surgical team, with post-operative care taking place on the thoracic surgical ward or in the intensive care unit if invasive monitoring or additional organ support were required.

Pre-operative smoking status was categorised in three groups: current smokers, defined as those who had smoked tobacco within 12 months leading up to surgery; ex-smokers, defined as those who had ceased smoking more than 12 months before surgery; and never smokers. Our ex-smoker definition is based on the low smoking relapse rate after 12 months of smoking cessation [25]. Smoking data were obtained from self-report and collected by healthcare workers including respiratory physicians, thoracic surgeons, and respiratory scientists.

### Data collection

Data extracted included baseline demographics, (age, sex, body mass index), type of operation performed, smoking exposure expressed in pack years, years since smoking cessation, pre-operative lung function (spirometry and carbon monoxide transfer factor (TLCO)), histological diagnosis, cancer stage (based on the International Association for the Study of Lung Cancer (IASLC) 7^th^ edition of Tumour Node Metastasis Classification of Malignant Tumors) [26], neoadjuvant treatment, post-operative length of stay, and complication rates.

The presence of COPD was determined on pre-bronchodilator spirometry, using a FEV1/FVC ratio of <0.7. Pre-bronchodilator, rather than post bronchodilator, measurements were used as approximately one quarter of the study population did not have post bronchodilator spirometry performed. COPD severity was defined using the Global Initiative for Obstructive Lung Disease (GOLD) criteria [27].

We compared pulmonary complications, overall complications, and length of stay between current smokers, ex-smokers and never smokers. We also compared pulmonary complications in those with and without a diagnosis of COPD. Pulmonary complications included: atelectasis, sputum retention, pneumonia, acute respiratory distress syndrome (ARDS), respiratory failure, air leak and empyema. Overall complications included: pulmonary complications, arrythmias, myocardial infarction, thromboembolic disease, stroke, transient ischaemic attack, renal failure, urinary tract infection, gastrointestinal bleed, ileus, wound infection / dehiscence and 30-day mortality (S1 Appendix).

### Statistical analysis

Statistical analysis was performed using SPSS v.25. Descriptive analyses were used to describe the study variables. Means and standard deviations were calculated for continuous variables, while proportions were used to describe categorical variables. For inferential analyses, chi-squared tests were used to compare smoking status and COPD status with other study variables including post-operative complications. Binary logistic regression was used to assess the strength of association, which yielded odds ratios (OR) and 95% confidence intervals (CI). Then multivariate logistic regression was used to adjust potential confounding variables (age, gender and BMI), which yielded adjusted OR (AOR) and 95% CI. Statistical significance was set at p<0.05.

## Results

### Baseline characteristics

Patient characteristics are shown in Table 1. A total of 1013 patients underwent curative-intent lobectomy or pneumonectomy. The mean age was 66 years (SD 12), with a male predominance (61%) and a mean BMI of 27 kg/m^2^ (SD 5).

**Table 1:** Patient characteristics.

Variables	Never smokers, n(%)		Current smokers, n(%)	p1	Ex-smokers, n(%)	p2	p3
Total participants^a^	111	314			362		
Age (years), Mean (±SD)	62.5 (16.9)	63.5 (10.3)		0.497	69.1 (9.5)	0.000	0.000
Male	30 (27.0)	205 (65.3)		0.000	254 (70.2)	0.000	0.175
BMI (kg/m^2^), Mean (±SD)	27.0 (5.1)	26.0 (5.3)		0.085	27.5 (4.5)	0.356	0.000
BMI categories	84	243			269		
Underweight (<18.5)	3 (3.6)	17 (7.0)		0.259	3 (1.1)	0.128	0.001
Healthy weight (18.5–24.9)	34 (40.5)	134 (55.1)		0.020	98 (36.4)	0.504	0.000
Overweight (25.0–29.9)	47 (56.0)	92 (37.9)		0.004	168 (62.5)	0.286	0.000
Primary operation^b^	111	314			362		
Lobectomy	105 (94.6)	281 (89.5)		0.109	318 (87.8)	0.043	0.502
Pneumonectomy	6 (5.4)	33 (10.5)		0.109	44 (12.2)	0.043	0.502
FEV1 (%), Mean (±SD)	94.3 (20.0)	81.2 (17.9)		0.000	83.8 (19.6)	0.000	0.082
FVC (%), Mean (±SD)	97.8 (18.1)	94.5 (15.6)		0.072	95.9 (17.0)	0.318	0.278
FEV1/FVC, Mean (±SD)	0.75 (0.1)	0.67 (0.1)		0.000	0.67 (0.1)	0.000	0.841
TLCO (%), Mean (±SD)	83.1 (16.4)	69.8 (16.6)		0.000	72.7 (18.3)	0.000	0.034
Pack-years, Mean (±SD)^c^		48.6 (24.7)			39.2 (25.5)		0.000
COPD present	22 (19.8)	180 (57.3)		0.000	205 (56.6)	0.000	0.856
Mild	11 (50.0)	65 (36.1)		0.204	81 (39.7)	0.341	0.469
Moderate	11 (50.0)	105 (58.3)		0.456	110 (53.9)	0.744	0.385
Severe	0 (0)	10 (5.6)		0.605	13 (6.4)	0.621	0.736
Stage^d^	108	307			356		
1	73 (67.6)	160 (52.1)		0.005	196 (55.1)	0.021	0.449
2	20 (18.5)	89 (29.0)		0.033	91 (25.6)	0.133	0.322
3	15 (13.9)	58 (18.9)		0.240	69 (19.4)	0.194	0.873
Histology^e^	111	314			362		
Adenocarcinoma	62 (55.9)	159 (50.6)		0.344	186 (51.4)	0.409	0.847
Squamous	8 (7.2)	94 (29.9)		0.000	114 (31.5)	0.000	0.662
Adenosquamous	0 (0)	8 (2.5)		0.090	13 (3.6)	0.043	0.435
Carcinoid	31 (27.9)	8 (2.5)		0.000	14 (3.9)	0.000	0.335
Others	10 (9.0)	45 (14.3)		0.151	35 (9.7)	0.836	0.259
Pathological diagnosis^f^	111	314			362		
Non small cell lung cancer	70 (63.1)	266 (84.7)		0.000	316 (87.3)	0.000	0.334
Small cell lung cancer	0 (0)	4 (1.3)		0.232	1 (0.3)	0.579	0.131

BMI: body mass index, FEV1: forced expiratory volume in 1 second, FVC: forced vital capacity, TLCO: transfer factor of the lung for carbon monoxide, COPD: chronic obstructive pulmonary disease.

p1 indicates comparison between never smokers and current smokers; p2 indicates comparison between never smokers and ex-smokers; p3 indicates comparison between current smokers and ex-smokers.

a: Smoking status was not recorded for 226 patients; hence they were excluded from the smoking status vs. complications analysis.

b: Totals: Lobectomy: 900, Pneumonectomy 113.

c: Pack-year data was missing for 6 current smokers and 9 ex-smokers.

d: Totals: Stage I: 559, Stage II: 261, Stage III: 193.

e: Totals: Adenocarcinoma: 521, Squamous: 279, Adenosquamous: 23, Carcinoid: 73, Others: 117.

f: Totals: Non-small cell lung cancer: 832, Small cell lung cancer: 7, Carcinoid 73.

### (i) Pre-operative smoking status

Three hundred and sixty-two patients (36%) were ex-smokers, 314 (31%) were current smokers and 111 (11%) were never smokers. Two hundred and twenty-six patients (22%) did not have their smoking status recorded in the database, hence they were excluded from the smoking status vs. complications analysis. Ex-smokers were older than never smokers (69 vs 63 years, p<0.001), with no age difference between current and never smokers (64 vs 63 years, p = 0.497). Compared to the never smokers, both current smokers and ex-smokers were more likely to be males (p<0.001). Current smokers had a higher mean pack-year smoking exposure history compared to ex-smokers (p<0.001).

### (ii) Pre-operative lung function and COPD diagnosis

Fifty-eight patients (6%) did not have lung function recorded in the database. Pre-operative spirometric values were higher in never smokers compared to both current and ex-smokers (Table 1), with mean percentage predicted FEV1s of 94%, 81% (p<0.001) and 84% (p<0.001), respectively. A similar pattern was seen with TLCO, with mean percentage predicted values in never smokers of 83%, compared to 70% (p<0.001) in current smokers and 73% (p<0.001) in ex-smokers. A pre-operative diagnosis of COPD was present in 20% of never smokers, compared to 57% of current smokers and 57% of ex-smokers. The never smokers had COPD of mild or moderate severity.

### Surgical and neoadjuvant treatment

Nine hundred patients (89%) underwent lobectomy, and the remaining 113 (11%) underwent pneumonectomy. Video-assisted thoracoscopy (VATS) was used in 111 (11%) patients. Additionally, 56 patients (6%) received neoadjuvant radiotherapy or chemotherapy, with no difference seen based on smoking status (3% of never smokers, 6% of current smokers and 6% of ex-smokers).

### Histology/Staging

Eight hundred and thirty-two patients (82%) had a pathological diagnosis of NSCLC, of which 521 (51%) were adenocarcinoma and 279 (28%) squamous cell carcinoma (SCC). NSCLC was more common amongst current smokers (85% vs 63%, OR 3.25, 95% CI 1.98–5.31, p<0.001) and ex-smokers (87% vs. 63%, OR 4.02, 95% CI 2.46–6.60, p<0.001) compared to never smokers. Similarly, SCC was more frequent in current smokers (30%, OR 5.5, 95% CI 2.58–1.7, p<0.001) and ex-smokers (32%, OR 5.92, 95% CI 2.79–12.6, p<0.001), compared to never smokers (7%). There was no difference in the frequency of adenocarcinoma in current or ex-smokers compared to never smokers. Conversely, carcinoid tumours were less common amongst current smokers (3% vs 28%, OR 0.07, 95% CI 0.03–0.15, p<0.001) and ex-smokers (4% vs 28%, OR 0.10, 95% CI 0.05–0.20, p<0.001) than among never smokers.

Most patients had Stage I disease (56%), with 26% having Stage II and 17% Stage III disease. Never smokers were more likely to be diagnosed with Stage I disease compared to both ex-smokers (68% vs. 55%, p = 0.021) and current smokers (68% vs. 52% p = 0.005). Conversely, current smokers were more likely to be diagnosed with stage II disease compared to never smokers (29% vs. 19%, p = 0.033). There was a trend towards higher rates of stage III disease in current and ex-smokers compared to never smokers.

### Post-operative complications

Post-operative complication rates are shown in Table 2. About a quarter (26%) of patients experienced a PPC, with 71 patients experiencing more than one. The rates of specific complications were: atelectasis 4%, sputum retention 4%, pneumonia 7%, ARDS 1%, respiratory failure 4%, air leak 12% and empyema 1%.

**Table 2:** Complication rates grouped by smoking status.

Variables	Never smokers, n(%)	Current smokers, n(%)	p1	Ex-smokers, n(%)	p2	p3
Total participants^a^	111	314		362		
All complications	50 (45.0)	146 (46.5)	0.792	161 (44.5)	0.916	0.599
Pulmonary complications^b^	19 (17.1)	85 (27.1)	0.036	88 (24.3)	0.113	0.412
Atelectasis	4 (21.1)	13 (15.3)	0.544	14 (15.9)	0.801	0.268
Sputum retention	6 (31.6)	20 (23.5)	0.474	11 (12.5)	0.061	0.026
Pneumonia	5 (26.3)	22 (25.9)	0.949	26 (29.5)	0.804	0.703
ARDS	0 (0)	5 (5.9)	N/A	2 (2.3)	N/A	0.089
Respiratory failure	4 (21.1)	14 (16.5)	0.629	12 (13.6)	0.611	0.140
Air leak	7 (36.8)	35 (41.2)	0.744	44 (50.0)	0.313	0.955
Empyema	1 (5.3)	3 (3.5)	1.000	5 (5.7)	0.988	0.932
Length of stay in days, Mean (±SD)	9.0 (7.1)	9.5 (6.6)	0.465	9.6 (6.9)	0.423	0.908

p1 indicates comparison between never smokers and current smokers; p2 indicates comparison between never smokers and ex-smokers; p3 indicates comparison between current smokers and ex-smokers.

ARDS: Acute respiratory distress syndrome.

a: Smoking status was not recorded for 226 patients; hence they were excluded from the smoking status vs. complications analysis.

b: Some participants had more than one pulmonary complication.

There were no differences in overall complication rates according to smoking status. PPCs were more frequent in current smokers compared to never smokers (27% vs 17%, p = 0.036), however there was no difference between current and ex-smokers (p = 0.412) or between never- and ex-smokers (p = 0.113). There were no differences in individual PPC rates or mean lengths of stay according to smoking status, other than increased sputum retention for current smokers compared to ex-smokers (p = 0.026).

As demonstrated in Table 3, those with a diagnosis of COPD had a higher frequency of both PPCs (65% vs 35%, AOR 1.76, 95% CI 1.02–2.41, p<0.01) and overall complications (60% vs 40%, AOR 1.53, 95% CI 1.17–2.01, p<0.01). Of the individual pulmonary complications, sputum retention (77% vs 23%, AOR 3.93, 95% CI 1.49–10.4), respiratory failure (78% vs 22%, AOR 3.21, 95% CI 1.13–9.12) and air leak (66% vs 34%, AOR 2.85, 95% CI 1.12–7.20) were more common in those with COPD. Mean length of stay was longer in those with COPD (10.3 vs 9.0 days, p<0.01). Table 4 shows that the complication rates in those with COPD did not differ between never and current smokers, apart from air leak being more common in current smokers (p<0.01). Table 5 shows that sputum retention was more common amongst current smokers compared to ex-smokers with COPD (AOR 10.9, 95% CI 1.58–74.9).

**Table 3:** Complication rates grouped by COPD status.

Variables	COPD	No COPD	Unadjusted analyses			Adjusted analyses		
			p	ORs	95% CIs	p	AORs*	95% CIs
Total participants^a^	500	455						
Post-operative complications	267 (59.7)	180 (40.3)	0.000	1.75	1.35–2.26	0.002	1.53	1.17–2.01
Pulmonary complications	159 (65.4)	84 (34.6)	0.000	2.06	1.52–2.79	0.001	1.76	1.02–2.41
Atelectasis	22 (56.4)	17 (43.6)	0.866	0.92	0.37–2.31	0.890	0.93	0.35–2.51
Sputum retention	34 (77.3)	10 (22.7)	0.010	3.25	1.30–8.13	0.006	3.93	1.49–10.4
Pneumonia	44 (68.8)	20 (31.3)	0.140	1.87	0.81–4.31	0.096	2.21	0.87–5.63
ARDS	4 (50.0)	4 (50.0)	0.706	0.75	0.17–3.35	0.637	0.69	0.15–3.22
Respiratory failure	28 (77.8)	8 (22.2)	0.033	2.83	1.07–7.49	0.029	3.21	1.13–9.12
Air leak	72 (66.1)	37 (33.9)	0.155	1.82	0.79–4.16	0.027	2.85	1.12–7.20
Empyema	7 (63.6)	4 (36.4)	0.823	1.17	0.30–4.51	0.901	0.90	0.18–4.43
Length of stay, Mean (±SD)	10.3 (7.4)	9.0 (6.9)	0.006	Mean difference (-1.29)	(-2.21) to (-0.38)			

OR: Odds Ratio, AOR: Adjusted Odds Ratio, 95% CI: 95% Confidence Interval, ARDS: Acute respiratory distress syndrome.

* Adjusted for: age, gender and BMI.

a: Lung function was not recorded in 58 patients; hence they were excluded from the COPD status vs. complications analysis.

**Table 4:** Complication rates grouped by smoking status (current and never-smokers) amongst COPD patients.

Variables	Current smokers with COPD	Never smokers with COPD	Unadjusted analyses			Adjusted analyses		
			p	ORs	95% CIs	p	AORs*	95% CIs
Total participants	180	22						
Post-operative complications	97 (88.2)	13 (11.8)	0.644	0.81	0.33–1.99	0.384	0.64	0.24–1.73
Pulmonary complications	58 (92.1)	5 (7.9)	0.364	1.62	0.57–4.60	0.777	1.18	0.38–3.64
Atelectasis	6 (66.7)	3 (33.3)	0.803	1.33	0.14–12.8	0.202	17.3	0.22–1375
Sputum retention	17 (85.0)	3 (15.0)	0.785	1.42	0.12–17.5	0.577	2.30	0.12–42.8
Pneumonia	13 (86.7)	2 (13.3)	0.844	1.30	0.10–17.7	0.737	1.71	0.07–39.3
ARDS	1 (100)	0 (0)	0.571	NA	NA	NA	NA	NA
Respiratory failure	9 (100)	0 (0)	0.086	NA	NA	NA	NA	NA
Air leak	24 (100)	0 (0)	0.003	NA	NA	NA	NA	NA
Empyema	2 (100)	0 (0)	0.429	NA	NA	NA	NA	NA
Length of stay, Mean (±SD)	10 (6.5)	10 (8.0)	0.976	Mean difference (-0.05)	(-3.01) to (2.92)			

OR: Odds Ratio, AOR: Adjusted Odds Ratio, 95% CI: 95% Confidence Intervals, ARDS: Acute respiratory distress syndrome.

* Adjusted for: age, gender and BMI.

**Table 5:** Complication rates grouped by smoking status (current and ex-smokers) amongst COPD patients.

Variables	Current smokers with COPD	Ex-smokers with COPD	Unadjusted analyses			Adjusted analyses		
			p	ORs	95% CIs	p	AORs	95% CIs
Total participants	180	205						
Post-operative complications	97 (49.2)	100 (50.8)	0.317	1.23	0.82–1.83	0.121	1.42	0.91–2.22
Pulmonary complications	58 (49.6)	59 (50.4)	0.464	1.18	0.76–1.82	0.343	1.26	0.78–2.03
Atelectasis	6 (46.2)	7 (53.8)	0.173	3.14	0.59–16.8	0.060	12.8	0.90–182
Sputum retention	17 (63.0)	10 (37.0)	0.024	4.68	1.17–18.7	0.015	10.90	1.58–74.9
Pneumonia	13 (39.4)	20 (60.6)	0.953	1.04	0.28–3.88	0.229	3.14	0.49–20.3
ARDS	1 (33.3)	2 (66.7)	0.952	1.08	0.08–14.4	0.754	1.67	0.07–42.2
Respiratory failure	9 (42.9)	12 (57.1)	0.328	1.95	0.51–7.49	0.144	3.68	0.64–21.1
Air leak	24 (43.6)	31 (56.4)	0.735	0.77	0.18–3.42	0.920	0.91	0.15–5.60
Empyema	2 (28.6)	5 (71.4)	0.943	0.93	0.14–6.23	0.215	5.31	0.38–74.1
Length of stay, Mean (±SD)	10 (6.5)	10.1 (7.6)	0.871	Mean difference (0.12)	(-1.30) to (1.53)			

OR: Odds Ratio, AOR: Adjusted Odds Ratio, 95%CI: 95% Confidence Interval, ARDS: Acute respiratory distress syndrome.

* Adjusted for: age, gender and BMI.

## Discussion

This study is the first to examine the association between smoking, COPD and post-operative complications following lung cancer resection in an Australian population. With 23 years of prospective follow up, this study is the longest single centre lung cancer resection cohort reported to date, as well as being one of the largest, world-wide. The study demonstrated a higher rate of PPCs following lung cancer surgery in current smokers compared to never smokers, which is comparable with previous literature [6, 24]. There was an increase in PPCs, overall complications and length of stay in those with COPD compared to those without, independent of smoking status. There was no significant difference in PPCs, overall complications or length of stay in ex-smokers compared to either current or never smokers.

There have been several studies examining smoking cessation and its effect on lung cancer surgery complication rates. Consistent with the results of our study, Fukui et al. demonstrated a higher PPC rate in smokers compared to never smokers in 666 patients who underwent lung cancer resections in a single centre in Japan [24]. In that study, however, differential results were noted in ex-smokers, with decreasing odds of a PPC associated with a longer duration of smoking cessation, and some reduction even after cessation for under a month. Lugg et al. examined a group of 462 patients undergoing lung cancer resections in a thoracic centre in the UK, and also found a higher rate of PPCs in smokers compared to never smokers [7]. There was a trend towards a lower PPC rate in ex-smokers, which was defined as those who had stopped any time prior to surgery. Nonetheless, they found no difference in PPCs in ex-smokers who had quit for more than or less than six weeks. Rodriguez et al. performed a case control study in 378 patients, examining current smokers and recently (within 16 weeks) reformed smokers, and found no difference in rates of pneumonia and atelectasis [20].

Part of the reason for the mixed evidence of the benefit of smoking cessation may lie in the different definitions used in the studies to characterise ex-smokers. We used a more parsimonious definition in our ex-smoker classification of one year of abstinence, as have other authors [28]. There is no consensus on the duration of smoking abstinence required in order to be defined as an ex-smoker. The most liberal definition of an ex-smoker is someone who has not smoked in the past 24 hours. We know that likelihood of quitting smoking on any given attempt is low, and that it takes multiple quit attempts to successfully abstain from smoking [29]. It is also known that smoking cessation for 12 months or more significantly reduces the risk of relapse [25]. Life insurance companies consider individuals as current smokers if they have smoked even one cigarette in the preceding 12 months [30]. The rate of smoking relapse after lung cancer resection is high. Cooley et al. studied a group of 84 ever-smokers who underwent lung cancer resection and found that, after one month, 18% had relapsed, increasing to 33% at two months and 42% at four months [31]. Ten participants who had quit before their diagnosis of lung cancer resumed smoking after surgery. Out of the ten, eight had abstained from cigarettes for a least a year prior to their lung cancer diagnosis. Even with our strict definition, some participants classified as ex-smokers in our study could have relapsed in the post-operative period. At the one-month mark in Cooley’s study, 13% admitted to a resumption of smoking, with a positive urine cotinine analysis (reflecting nicotine intake), increasing the proportion to 18%. The above-mentioned studies by Fukui, Lugg and Rodriguez all relied on self-report, where it is possible that some of the participants who reported they were ex-smokers (especially those who had quit recently), were still smoking, skewing the results. Our definition of an ex-smoker may make it less likely to find differences between never and ex-smokers, although more likely to find differences between current and ex-smokers.

Similarly, there is no universally accepted definition of a PPC, resulting in rates ranging from 5% in a study that characterised PPC as atelectasis requiring bronchoscopy and or pneumonia (Rodriguez et al) to 32% in another study that defined a PPC as hypoxia, pneumonia, atelectasis and or uncontrolled sputum production (Fukui et al) [20, 24]. It is possible that studies with a more inclusive PPC definition would be better powered to find a difference in complication rates. Compared to the above studies our definition of a PPC was more comprehensive, and hence a better representation of the true respiratory complication rate and the effect smoking status has on this.

Despite the mixed evidence of its value in the perioperative period, smoking cessation is still beneficial in early stage lung cancer, with reduced risk of cancer recurrence, lower rates of development of a secondary primary tumour and improved long term survival in those who quit [32]. Long term survival is thought to be driven by a reduction in both cardiorespiratory and cancer-related deaths. Indeed, a new lung cancer diagnosis may be an effective time to employ smoking cessation strategies, with a retrospective analysis showing that an intensive smoking cessation program prior to lung cancer resection achieved smoking cessation rates of just over 50% at 24 months [33]. For these reasons, we believe that smoking cessation should still be actively pursued in the preoperative period.

In concordance with our results, several previous studies have shown a higher frequency of PPCs in patients with COPD [16–19]. The COPD incidence of 57% in smokers or ex-smokers in our study population is comparable with that in other lung cancer cohorts [12, 34]. Given the increased perioperative risk associated with COPD, the question is raised as to whether post-operative outcomes can be improved through peri-operative optimisation of COPD management. Our management of patients with lung cancer has always been multidisciplinary, involving regular meetings between respiratory physicians, thoracic surgeons, oncologists, radiologists, pathologists, and radiotherapists. This has included regular referral of patients to respiratory physicians for pre-operative maximisation of COPD therapy, as well as consensus decision-making regarding the appropriateness of surgery as the treatment modality. Key aims of COPD management include optimisation of function and prevention of exacerbations. Not only are exacerbations associated with increased mortality [35], but the risk of exacerbation is increased post thoracic surgery [36], and those with reduced lung function have poorer surgical outcomes [37]. Thus, therapies that improve lung function and reduce the risk of exacerbation could be expected to be beneficial in the perioperative setting.

Long-acting bronchodilators improve lung function and symptoms, and decrease exacerbations [38]. There is some evidence that inhaled long-acting muscarinic antagonists (LAMAs) and long-acting beta agonists (LABAs) reduce post-operative complications, however studies have mostly been retrospective in nature, with small numbers of participants [39–41]. The combination of LAMA and LABA has been shown to improve lung function, symptoms and exacerbation rates more than bronchodilator monotherapy [42]. Makino et al. performed a retrospective analysis of 33 patients, comparing combination LAMA/LABA with bronchodilator monotherapy, demonstrating a reduction in the rate of post-operative pneumonia in the combination group, but no difference in any other pulmonary or cardiovascular complication [43]. Inhaled corticosteroids (ICS) are recommended in patients with recurrent exacerbations of their COPD, concomitant asthma, and/or elevated serum eosinophil levels [27]. Bölükbas et al. compared LAMA/LABA vs. LAMA/LABA/ICS therapy prospectively in a group of 46 patients newly diagnosed with both lung cancer and moderate to severe COPD [44]. After one week of treatment, lung function improved in both groups, with more patients in the triple therapy group having a greater than 10% improvement in FEV1 as well as a decrease in GOLD COPD severity class. A statistically significant reduction in PPCs was seen in the triple therapy group as well, driven by reductions in pneumonia and sputum retention. We have found no other trials looking at ICS therapy or dual versus mono bronchodilator therapy in patients undergoing lung cancer resection. Despite a lack of high quality evidence it would seem appropriate to maximise bronchodilatation in patients with COPD in the peri-operative period, and to consider the addition of ICS in those with moderate to severe COPD, an allergic phenotype, or an elevated serum eosinophil count.

Pre-operative pulmonary rehabilitation or “prehabilitation” may also reduce perioperative risk. Pulmonary rehabilitation programs have been shown to improve symptoms and quality of life and to reduce exacerbations [45, 46]. Although most programs are 6–8 weeks in duration, there is evidence that shorter duration programs can be beneficial. Mujoiv et al. demonstrated that a 2–4 week period of pre-operative pulmonary rehabilitation improved lung function in a group of patients with NSCLC and COPD awaiting surgery, although bronchodilator therapy was included as part of the program, which may have contributed to this improvement [47]. In a randomised controlled trial (n = 101) of a one week high intensity inpatient exercise program prior to lobectomy, Lai et al demonstrated a reduction in PPCs and length of stay in the exercise group [48]. A meta-analysis showed that inspiratory muscle training reduces PPCs and length of stay in cardiac, pulmonary, and abdominal surgical patients [49], and a recent randomised controlled trial demonstrated similar benefits in a group of patients with lung cancer [50]. Many of the studies examining the impact of prehabilitation on complications of lung cancer resection have included unselected populations, rather than those who have comorbid COPD. A meta-analysis performed by Li et al. included three randomised controlled trials looking at PPCs in those with COPD and lung cancer. Whilst there was an overall reduction in length of stay, there was only a trend to a reduction in PPCs, that did not reach statistical significance, however numbers were small [51]. We believe it would be reasonable to recommend exercise therapy prior to surgery if this does not delay surgery. More studies targeting the COPD population specifically are required to determine the type and duration of the exercise program.

The proportion of never smokers with COPD in our study (just on 20%) is reflective of the literature [12, 52]. The airflow obstruction in the never smokers in our study could relate to second-hand smoke exposure, air pollution, or asthma. We were unable to determine the exact aetiology as comorbid conditions and environmental exposures were not recorded in the database. There is a possibility that airflow obstruction was over diagnosed in older patients, as we used a fixed 0.7 cut off for FEV1/FVC ratio, rather than an age adjusted lower limit of normal [52]. As the ex-smokers in our study were older than the current smokers, this may have led to a higher complication rate in the ex-smokers. It is possible this may have contributed to the lack of difference in complications seen between current and ex-smokers.

There are a few limitations of our study. The first is that we were not able assess the impact of a shorter period of smoking cessation on complication rates. Although our definition of a current smoker allows us to characterise more accurately those who were probably still smoking, it does not allow us to make a granular assessment of the impact of smoking cessation in the days to weeks prior to surgery. Using a shorter period of abstinence to define quitting as well as using objective testing for cigarette use would be a way to examine this in the future. The second limitation is the use of pre-, rather than post-bronchodilator spirometry, to define COPD. This may have overestimated the number of patients who truly had COPD, although it is not uncommon to use pre-bronchodilator spirometry to diagnose COPD if post-bronchodilator spirometry is not available. The third limitation is that we do not have data on pre-existing comorbidities that may have increased the post-operative complication rate. In our cohort of smokers and in those with COPD, cardiovascular disease would likely have been the most significant comorbidity. This may have contributed to the overall complication rate but is unlikely to significantly impact the pulmonary complication rate. Notwithstanding these limitations, our study is an important addition to the literature, given our large study population and comprehensive data set. Complications were clearly defined and prospectively recorded. Spirometry was available on nearly all our patients and hence we were able to comment usefully on the likelihood of a concurrent diagnosis of COPD, its severity, and the impact of the COPD diagnosis on outcomes.

## Conclusion

Smoking and COPD are both associated with a higher rate of PPCs post lung cancer resection. COPD, independent of smoking status, is also associated with an increased overall post-operative complication rate and length of hospital stay. Smoking cessation should be pursued in the preoperative period, however an emphasis on COPD treatment optimisation, rather than smoking cessation in isolation, may maximise post-operative outcomes. Future studies should assist in determining the most appropriate peri-operative program of smoking cessation, “prehabilitation” and pharmacotherapy to maximise outcomes in patients undergoing lung cancer surgery.

## Supporting information

S1 Appendix Smoking, COPD, lung cancer. (DOCX)
