# Supplementary material for: Impact of smoking status and chronic obstructive pulmonary disease on pulmonary complications post lung cancer surgery
Source: PLoS One. 2022 Mar 29;17(3):e0266052. doi: 10.1371/journal.pone.0266052 (PMC8963579; doi:10.1371/journal.pone.0266052)
Supplement: S1 Appendix — (DOCX) [file pone.0266052.s001.docx]

**S1 Appendix**

**Definition of complications**

**Respiratory**

**Atelectasis**

Post-operative radiological evidence of plate atelectasis or focal areas of collapse usually associated with hypoxaemia plus or minus fever.

**Sputum retention**

Difficulty with the clearance of respiratory secretions such that mini-tracheostomy, formal tracheostomy, nasotracheal suction or bronchoscopic clearance is required.

**Pneumonia**

Radiological consolidation, fever, and clinically infected sputum or positive sputum culture.

**ARDS**

Bilateral pulmonary opacification emanating from the hilar regions in the absence of demonstrated infection or cardiac failure.

**Respiratory failure**

Ventilation for more than 48 hours post-operative, or re-intubation, or a PCO_2_ of greater than 50 mmHg for more than 4 hours.

**Air leak**

Clinically evident air leak for more than 7 days duration.

**Empyema**

Clinical or culture positive infection in the pleural space post-operatively.

**Cardiac**

**Atrial fibrillation**

ECG evidence of atrial fibrillation or flutter occurring post-operatively, which was not present pre-operatively.

**VF**

Electrocardiographically evident ventricular fibrillation or sustained ventricular tachycardia.

**AMI**

Clinical and electrocardiographic, or enzymatic evidence of new myocardial infarction.

**Thromboembolic**

**DVT**

Duplex ultrasonographic, or radiological (venogram), or nuclear evidence of a deep venous thrombosis.

**PE**

VQ scan, CT or angiographic evidence of pulmonary embolism.

**Anastomotic leak**

Clinical or radiographic evidence of an anastomotic leak at any site.

**Renal**

**UTI**

Demonstration of a significant number of bacteria in the urine (greater than 10,000 organisms per mL.) in association with a clinically evident urinary tract infection.

**Renal failure**

In patients with no renal impairment pre-operatively: elevation in creatinine to greater than twice baseline, or the need for dialysis in the post-operative period. In those patients with established renal impairment: an increase in the creatinine to greater than 200 micromol/L, or the need for dialysis post-operatively when not previously required.

**Cerebral**

**TIA**

Clinical evidence of a neurological deficit of less than 24 hours duration post-operatively.

**Stroke**

Clinical evidence of a neurological deficit of greater than 24 hours duration post-operatively.

**Gastrointestinal**

**Bleed**

Haematemesis of greater than 500 mL, (fresh or coffee grounds), melena or bright rectal blood loss, or a haemoglobin drop of greater than 20 g/L attributed to GI losses.

**Ileus**

Intestinal atony of greater than 72 hours duration.

**Wound**

**Infection**

Clinical evidence of wound erythema induration or suppuration associated with fever with or without the presence of a positive culture.

**Disruption**

Spontaneous breakdown of the wound with or without apparent infection.

**Other**

Any significant post-operative complication which does not readily fit into the prior classifications.

**Death**

Any death occurring within 30 days of the operative procedure or at any time during the hospitalisation during which the operative procedure was undertaken.
